# Group II Metabotropic Glutamate Receptors Reduce Apoptosis and Regulate BDNF and GDNF Levels in Hypoxic-Ischemic Injury in Neonatal Rats

**DOI:** 10.3390/ijms23137000

**Published:** 2022-06-23

**Authors:** Ewelina Bratek-Gerej, Apolonia Ziembowicz, Elzbieta Salinska

**Affiliations:** Department of Neurochemistry, Mossakowski Medical Research Institute, Polish Academy of Sciences, 02-106 Warsaw, Poland; aziembowicz@imdik.pan.pl (A.Z.); esalinska@imdik.pan.pl (E.S.)

**Keywords:** hypoxia-ischemia, birth asphyxia, group II metabotropic glutamate receptors, LY379268, NAAG, neuroprotection, apoptosis, neurotrophins

## Abstract

Birth asphyxia causes brain injury in neonates, but a fully successful treatment has yet to be developed. This study aimed to investigate the effect of group II mGlu receptors activation after experimental birth asphyxia (hypoxia-ischemia) on the expression of factors involved in apoptosis and neuroprotective neurotrophins. Hypoxia-ischemia (HI) on 7-day-old rats was used as an experimental model. The effects of intraperitoneal application of mGluR2 agonist LY379268 (5 mg/kg) and the specific mGluR3 agonist NAAG (5 mg/kg) (1 h or 6 h after HI) on apoptotic processes and initiation of the neuroprotective mechanism were investigated. LY379268 and NAAG applied shortly after HI prevented brain damage and significantly decreased pro-apoptotic Bax and HtrA2/Omi expression, increasing expression of anti-apoptotic Bcl-2. NAAG or LY379268 applied at both times also decreased HIF-1α formation. HI caused a significant decrease in BDNF concentration, which was restored after LY379268 or NAAG administration. HI-induced increase in GDNF concentration was decreased after administration of LY379268 or NAAG. Our results show that activation of mGluR2/3 receptors shortly after HI prevents brain damage by the inhibition of excessive glutamate release and apoptotic damage decrease. mGluR2 and mGluR3 agonists produced comparable results, indicating that both receptors may be a potential target for early treatment in neonatal HI.

## 1. Introduction

Neonatal hypoxic-ischemic (HI) encephalopathy, which occurs in 1–6 out of 1000 births, causes a major number of perinatal mortality and chronic disability in newborns worldwide [1,2]. The surviving infants develop various degrees of neurologic deficits that range from slight cognitive impairments to severe cases of cerebral palsy [3].

mGlu2/3 agonists are reported to be neuroprotective in several in vivo models of brain ischemia and hypoxia-ischemia [4,5,6,7,8]. In contrast to growing data on the mGlur2/3 neuroprotective role, no clear information is available on the mechanisms involved.

Although a reduction in glutamate release by activation of presynaptic mGluR2/3 receptors is an attractive hypothesis, there is ample evidence, particularly in vitro, to suggest that regulation of glutamate release may not always account for the observed neuroprotective effects of mGlu2/3 receptor agonists. Moreover, it was shown that in young animals, mGluR2/3 are present in post-synaptic membranes and that their activation results in a hyperpolarizing response [9,10,11].

Necrosis and oxidative stress-mediated apoptosis are involved in the course of HI-induced brain injury [12,13,14]. Oxidative stress-mediated mitochondrial injury after ischemia-reperfusion (I/R) changes the Bcl-2 family proteins balance, resulting in Bax-dependent mitochondrial outer membrane permeabilization [15,16]. This induces cytochrome C, apoptosis-inducing factor (AIF), Endo G, and Smac/DIABLO (second mitochondria-derived activator of caspase/direct IAP binding protein with a low pI) leakage into the cytosol, activation of caspases, and development of apoptosis [17,18].

Apoptosis induced by HI can take two paths, caspase-dependent and -independent, and mitochondrial serine protease HtrA2/Omi is involved in both. HtrA2 is released into the mitochondrial matrix, and cytosol regulates cell death through its interaction with apoptotic and autophagic signaling pathways [19]. It was shown that Omi/HtrA2 is closely related to the pathogenesis of neurological diseases, such as neurodegeneration and hypoxic-ischemic brain damage [20,21]. 

Hypoxia-inducible factor-1α (HIF-1α) is considered a regulator of both pro-survival and pro-death responses in the central nervous system. However, in severe or sustained hypoxic conditions such as neonatal HI, cumulative HIF-1 activation may result in pro-death responses [22]. On the other hand, it was shown that HIF-1α induces the expression of pro-survival genes (EPO, VEGF) and can decrease the expression of pro-apoptotic Bax/Bid/Bad [22,23].

Given the key role of neurotrophic factors in neuroprotection, an interaction between neurotrophic factors and the mGlu2/3 receptor signaling system has been suggested. HI reduces BDNF expression the neonatal brain, whereas the expression of GDNF significantly increases [24,25,26,27]. It was shown that activation of mGluR2/3 present in neurons, astrocytes, or oligodendroglia cells may stimulate the production of neurotrophic factors such as transforming-growth factor-1 (TGF-1) [28], brain-derived neurotrophic factor (BDNF) [29,30], and glial-derived neurotrophic factor (GDNF) [31].

Studies show that BDNF protects against HI-induced brain damage by inhibition of apoptosis, its anti-inflammatory and anti-toxic effects, and the promotion of neural regeneration [32] (Chen et al., 2013). GDNF, another important neurotrophin, inhibits caspase-3 activation and subsequently antagonizes neuronal apoptosis after HI [24,33]. Recent studies indicated that GDNF treatment can improve neurological outcome and reduce inducible nitric oxide synthase, TNF-α, and nitric oxide production [33].

Our previous experiments showed that activation of group II mGluRs up to 24 h before HI significantly reduces HI-evoked brain injury, oxidative stress, and apoptosis [8,34,35]. We showed also that application of mGluR2 or mGluR3 agonists within the therapeutic window for birth asphyxia (up to 6 h after HI) reduced weight loss in the ischemic hemisphere and HI-induced oxidative stress [7].

Taking into consideration the potential therapeutic application of mGluR2/3 agonists, the more detailed examination of the mechanisms of observed neuroprotection seems justified. This study aimed to investigate the effect of group II mGluR activation after experimental HI on rat pups on the expression of factors initiating apoptotic processes and neuroprotective neurotrophins.

## 2. Materials and Methods

### 2.1. Ethics Approval and Consent for Participation

All experiments described in this study were approved by the Second Local Ethical Committee based in Warsaw, Poland, and were performed following Polish governmental regulations (Dz.U.2015. poz.266) and the European Community Council Directive 2010/63/EU. Each experiment was performed on 3 different litters (10–12 rats per litter), and animals were randomly selected for experimental groups (2–3 animals from each litter). All surgeries were performed under isoflurane anesthesia, and all efforts were made to minimize animal suffering and the number of animals used (total number of animals used: 150). The mortality rate did not exceed 5%.

### 2.2. Experimental Hypoxia-Ischemia

Seven-day-old rat pups were anesthetized with isoflurane (4% for induction, and 1.5–2.0% for maintenance, in 0.6:1 nitrous oxide and oxygen), and the left common carotid artery was exposed and cut off between double silk ligatures (according to Rice et al., 1981) [36]. Pups were returned to their dams and recovered for 1 h at 37 °C. After recovery, pups underwent 75 min hypoxia (7.5% oxygen in nitrogen) in a humidified chamber at 35 °C. In this experimental model, unilateral carotid ligation followed by timed hypoxia exposure induces hypoxic-ischemic damage in the ipsilateral brain hemisphere. The opposite hemisphere remained undamaged and was used as an internal control. After hypoxic treatment, the rats were returned to their mothers and were maintained in climate-controlled rooms (21 to 24 °C; 50% to 55% humidity) with diurnal lighting (12:12-h light/dark photoperiod). Sham-operated rats were used as controls. The condition of the animals, which remained in the experiment for fourteen days, was evaluated twice per day and, if necessary, an anesthetic was applied locally.

### 2.3. Drug Application

In presented experiments, group II mGluR agonists were applied at the first and last hour of a narrow therapeutic window typically lasting approximately 6 h after the insult [37,38,39,40].

mGluR2 agonist LY379268 (5 mg/kg) or the specific mGluR3 agonist NAAG (N-acety-laspartyl-glutamate; 5 mg/kg) were administered intraperitoneally (i.p.) 1 h or 6 h after hypoxia-ischemia (HI). The dose of agonists was determined based on previously published findings [7,41]. Sham-operated and HI control rats were injected with saline.

### 2.4. TTC Staining

The chosen animals from each group (n = 4–5 per group) were sacrificed 7 days after the HI for an evaluation of the brain infarct area. Anesthetized rats were decapitated, and their brains were separated on ice. Each brain was cut into 2 mm thick sections. The sections were then placed in 2% TTC with a foil cover and incubated in an incubator at 37 °C for 15–30 min, making sure that the brain tissues were evenly contacted with the staining solution.

A camera and image analysis system was used for image acquisition and analysis. Infarct volume percentage (%) = (the calibrated infarct volume/volume of the contralateral hemisphere) × 100%.

### 2.5. Western Blot Analysis of Pro- and Antiapoptotic Protein Expression

The expression of anti-apoptotic protein Bcl-2 and pro-apoptotic proteins Bax and HtrA2/Omi were determined by western blot.

Tissues from both hemispheres were collected 2 h after agonists injection and homogenized separately in PBS buffer containing 10 mM EGTA, 10 mM EDTA, 0.1 mM PMSF, 100 mM NaCl, and protease inhibitor mixture. Protein concentration was determined by the Bradford method. The prepared samples (50 µg protein per 25 µL of homogenate) were subjected to SDS-polyacrylamide gel electrophoresis, and the separated proteins were transferred to nitrocellulose membranes. Membranes were incubated in 20 mM TBS/0.05% Tween/5% skimmed milk solution for 60 min, washed, and then incubated overnight at room temperature with selected primary antibodies specific for the proteins analyzed (Bcl-2, rabbit polyclonal antibody, Cell Signaling, Danvers, MA, USA; Bax, rabbit polyclonal antibody, Cell Signaling; HtrA2/Omi, rabbit polyclonal antibody, ThermoFisher, Waltham, MA, USA; β-actin, goat polyclonal antibody, Abcam, Cambridge, UK) at dilutions of 1:100 and 1:500 for β-actin. After rinsing, membranes were incubated for 1 h with appropriate alkaline phosphatase-conjugated secondary antibodies (Sigma-Aldrich, St. Louis, MO, USA). Protein bands were visualized using Vector Blue Alkaline Phosphatase Substrate Kit (Vector Laboratories, Burlingame, CA, USA), scanned using Image Scanner III (GE Healthcare, Chicago, IL, USA), and measured densitometrically using ImageQuant. Changes in protein expression are shown as the percentage of control. β-Actin (Abcam, diluted 1:500) was used as an internal standard.

### 2.6. Determination of the Expression of Selected Proteins and Neurotrophic Factors by ELISA

Tissues from the right and left hemispheres were collected 2 h after agonists injection and homogenized separately in 50 mM potassium orthophosphate containing 1 mM EDTA (pH 7.0). Homogenates were incubated in RIPA buffer for 1 h at 4 °C; then, the samples were centrifuged for 10 min at 10,000× *g* at 4 °C. After centrifugation, the lysates were transferred to new, cooled 1.5 mL polyethylene tubes. Protein concentration was measured (a Bradford method), and samples were frozen at −80 °C for further determination.

Expression of hypoxia-inducible factor 1-alpha (HIF-alpha), as well as BDNF and GDNF, was determined using ELISA kits (BDNF Elisa Kit and GDNF Elisa Ki, Abcam; HIF-1α ELISA Kit MyBioSource, San Diego, CA, USA) according to the manufacturer’s instructions.

### 2.7. Statistical Analysis

Results are expressed as the mean values ± SEM of each experimental group. Statistical analysis was performed using one-way ANOVA with Dunnett’s post hoc test for significant differences between groups (GraphPad Prism 5, San Diego, CA, USA). Differences were considered statistically significant when the *p*-value was less than 0.05.

## 3. Results

### 3.1. LY379268 and NAAG Reduce HI Evoked Brain Damage

**Figure 1:**
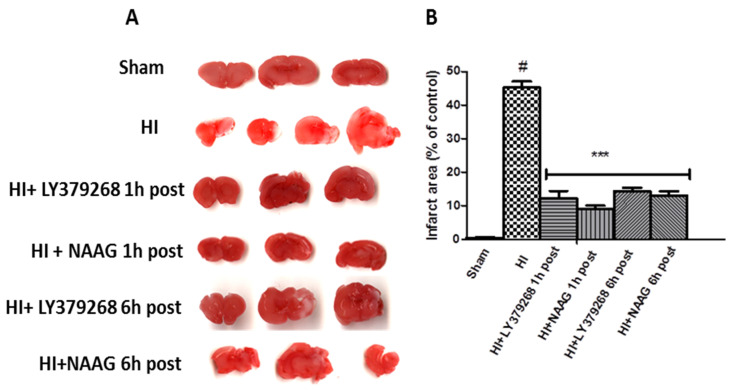
(**A**) Application of LY379268 or NAAG after hypoxia-ischemia (HI) reduces the infarct area in the ipsilateral hemisphere. Representative pictures of TTC staining of the infarct areas in the brains of rats; (**B**) Quantification of the infarct area after HI revealed by TTC staining. The number of animals per group n = 4–5. Results are presented as the mean values ± SD, *** *p* < 0.001—significantly different from the HI group; #—significantly different from the sham. The brains were analyzed 7 days after HI.

HI resulted in brain tissue damage in the ipsilateral brain hemisphere. In the present study, the evaluation of the infarct area using TTC staining showed that HI produced 46% infarction of the ipsilateral hemisphere (Figure 1).

LY379268 applied 1 or 6 h after HI significantly decreased the area of infarction to 11.6% and 14.5%, respectively (*p* < 0.001). NAAG applied 1 or 6 h after HI also significantly reduced the area of infarction to 9.2% and 12.3%, respectively (*p* < 0.001). The infarct areas measured after application of LY379268 or NAAG 1 h and 6 h after HI were not statistically different.

### 3.2. The Effect of Post-Treatment with mGluR2/3 Agonists on the Expression of Anti- and Pro-Apoptotic Factors in Response to HI

**Figure 2:**
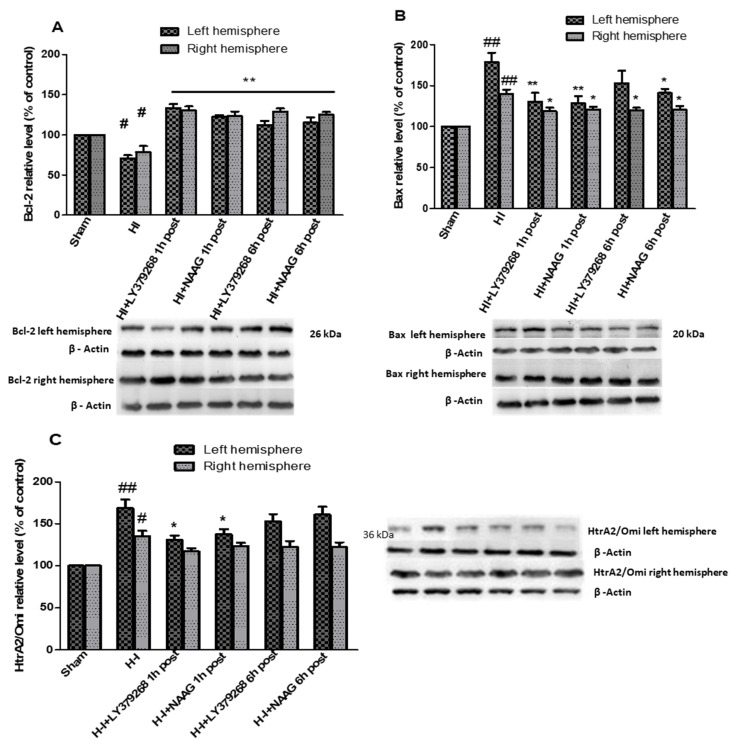
The effect of mGluR2 and mGluR3 agonists application on HI induced changes in Bcl-2 (**A**), Bax (**B**) and HtrA/Omi (**C**) expression in the brain. Results are presented as the mean ± SEM, n = 6–8; statistically significant differences: * *p* < 0.05. ** *p* < 0.01 compared to HI; ## *p* < 0.005, # *p* < 0.01 compared to the sham-operated group.

Hypoxia-ischemia in the immature brain changed the expression of a significant number of proteins. Levels of pro- and anti-apoptotic factors after HI were markedly changed in both the ipsilateral and contralateral hemispheres, probably as a response not only to ischemia but also 75 min hypoxia. We observed an HI-induced decrease in Bcl-2 expression in both hemispheres to 70.5% and 78.5% of the control in the left and right hemispheres, respectively (*p* < 0.01 in left, *p* < 0.05 in right, respectively; Figure 2A).

Application of each of examined group II mGluR agonists not only inhibited the decrease in Bcl-2 level but also resulted in a significant increase in both hemispheres. LY379268 applied 1 h after HI increased Bcl-2 expression to 133.5% and 130% of the control in the left and right hemisphere, respectively (*p* < 0.005 compared to control and HI group). LY379268 application 6 h after HI increased Bcl-2 expression in the left to the level comparable with control (112%, *p* > 0.07 compared to the control) but statistically lower compared to the level measured when LY379268 was applied 1 h after HI.

However, application of LY379268 6 h after HI resulted in a significant increase in Bcl-2 expression in the right hemisphere, reaching the level of 129% of the control (*p* < 0.01); this increase was almost identical to that observed in the right hemisphere after LY379268 application 1 h after HI.

Treatment of animals with NAAG 1 h after HI also inhibited the decrease in Bcl-2; we observed a significant increase in Bcl-2 expression in both hemispheres to 122% and 123% of the control for the left and right hemispheres, respectively (*p* < 0.001 compared to the control and HI group). Application of NAAG 6 h after HI also resulted in a significant increase in Bcl-2 levels to 115% of the control in the left hemisphere and 125% in the right hemisphere. There were no statistically significant differences between Bcl-2 expression observed in groups injected with NAAG 1 h or 6 h after HI.

Hypoxia-ischemia significantly increased pro-apoptotic Bax expression in both hemispheres (Figure 2B). Application of mGluR2 agonist LY379268 1 h after HI decreased enhanced expression of pro-apoptotic Bax in both hemispheres from 187% and 145% to 138% and 122% of the control, in left and right hemispheres, respectively (*p* < 0.05 compared to the HI and sham groups). Application of LY379268 6 h after HI decreased Bax levels to 152% and 116% of the control in both hemispheres; however, this decrease was statistically significant only in the right hemisphere (*p* < 0.05 compared to the HI group). mGluR3 agonist NAAG, applied 1 h after HI significantly reduced Bax expression in both hemispheres to 126% and 117% of the control, respectively (*p* < 0.001 and *p* < 0.02 in left and right hemisphere compared to the HI groups, respectively).

Application of NAAG 6 h after HI decreased Bax levels in both hemispheres to 146% and 116% of the control (*p* < 0.05 compared to the HI). However, the decrease in Bax expression measured in experimental groups injected with NAAG 1 h or 6 h after HI was not statistically different.

The expression of pro-apoptotic serine protease HtrA2/Omi increased significantly after HI in left and right hemispheres to 169% and 135% of the control, respectively (*p* < 0.005 in left, *p* < 0,01 in right hemisphere) (Figure 2C). LY379268 applied 1 h after HI decreased HtrA2/Omi expression only in the left hemisphere to 131% of the control (*p* < 0.05 compared to the HI group); however, LY379268 application 6 h after HI did not result in a statistically significant decrease in HtrA2/Omi expression (152%, *p* > 0.05). Application of LY379168 in both examined times did not significantly reduce HI-induced increase in HtrA2/Omi expression in the right hemisphere.

Similar to the mGluR2 agonist, mGluR3 agonist NAAG decreased HtrA2/Omi expression only in the left hemisphere when injected 1 h after HI (137%, *p* < 0.05). We did not observe a statistically significant decrease in HtrA2/Omi expression either in the left hemisphere after NAAG application 6 h after HI or in the right hemisphere after NAAG application at both examined times.

### 3.3. Effect of mGluR2/3 Agonists Application on HIF-Alpha Expression after Hypoxia-Ischemia

**Figure 3:**
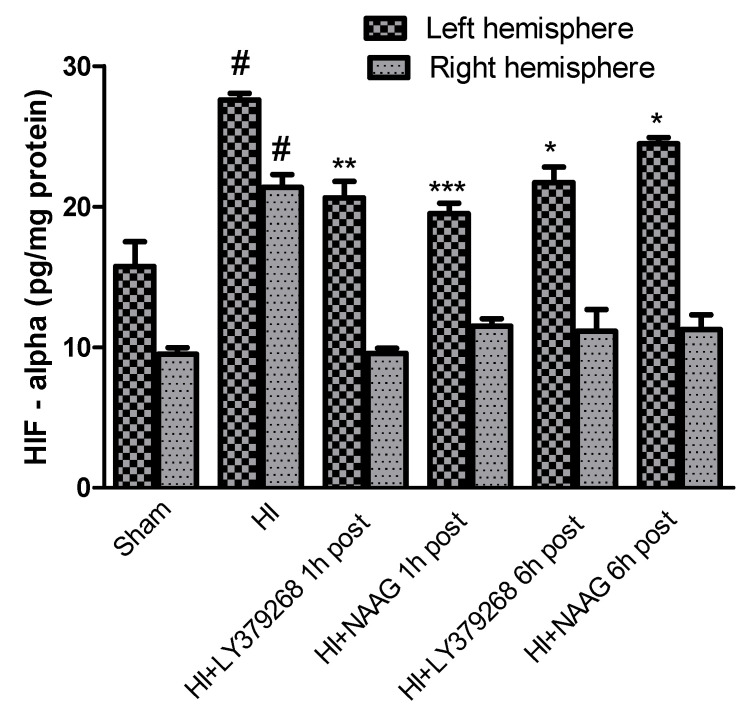
The effect of LY379268 or NAAG application 1 h or 6 h after HI on HIF-1α concentration. Results are presented as the mean ±SEM, n = 5; statistically significant differences: * *p* < 0.01; ** *p*< 0.005, *** *p* < 0.001—significantly different from the HI group; # *p* < 0.005 significantly different from the sham operated group.

The concentration of HIF-1α in brain samples isolated from sham-operated rats was 15.7 pg/mg protein in the left hemisphere and 9.5 pg/mg protein in the right hemisphere. Hypoxia-ischemia significantly increased the concentration of HIF-1α to 27.6 pg/mg protein and 21.3 pg/mg protein in the left and right hemispheres, respectively (*p* < 0.005 for the left and right hemispheres, respectively) (Figure 3).

Application of LY379268 1 h or 6 h after HI significantly reduced HIF-1α expression in the left hemisphere to 20.6 pg/mg protein and 21.7 pg/mg, respectively (*p*< 0.005 and *p* < 0.01, respectively).

NAAG applied 1 h or 6 h after HI also significantly decreased the concentration of HIF-1α in the left hemisphere to 19.5 and 24.4 pg/mg protein, respectively (*p* < 0.001 and *p* < 0.01, respectively). The application of both agonists decreased HIF-1α in the right hemisphere to the control level regardless of the time of administration.

The observed effects of LY379268 and NAAG were comparable and did not differ statistically.

### 3.4. The Effect of mGluR2/3 Agonists on the Changes in Neurotrophic Factors Expression after Hypoxia-Ischemia

**Figure 4:**
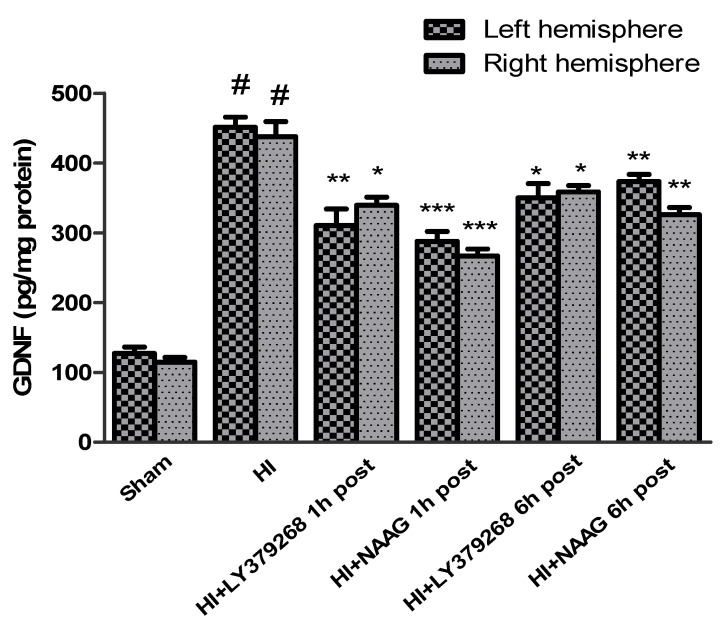
Effect of mGluR2 or mGluR3 agonists injection on GDNF level. Results are presented as mean ± SEM, n = 6–8; statistically significant differences: * *p* < 0.05, ** *p* < 0.01, *** *p* < 0.005—different from H-I; # *p* < 0.001—different from sham operated group.

The concentration of GDNF in brain samples from sham groups ranged from 127.3 pg/mg protein in the left hemisphere to 114 pg/mg protein in the right hemisphere. Hypoxia-ischemia induced a fourfold increase in the GDNF expression in both hemispheres compared to the sham group: 451.3 pg/mg protein in the left and 437.6 pg/mg protein in the right hemisphere (*p* < 0.0001) (Figure 4).

LY379268 applied 1 h after HI decreased GDNF expression in both hemispheres to 310 pg/mg and 340 pg/mg protein in the left and right hemispheres, respectively (*p* < 0.01 and *p* < 0.05 compared to the HI group, respectively). LY379268 application 6 h after HI decreased GDNF expression in both hemispheres to 350 pg/mg and 358 pg/mg protein in the left and right hemispheres, respectively (*p* < 0.05 compared to the HI group).

NAAG applied 1 h after HI also decreased GDNF expression in both hemispheres to 288 pg/mg protein in the left and 267 pg/mg protein in the right hemispheres, respectively (*p* < 0.005 compared to HI for both groups). Application of NAAG 6 h after HI also reduced GDNF expression, to 373 pg/mg protein in the left hemisphere and 326 pg/mg protein in the right hemisphere (*p* < 0.01 compared to the HI group).

The effect of both agonists’ application on GDNF expression was not statistically different; however, the decrease of GDNF expression after application of both agonists 6 h after HI was significantly lower than when they were applied 1 h after insult.

The concentration of BDNF measured in brain samples isolated from sham-operated rats was 22.3 pg/mg protein in the left hemisphere and 23.5 pg/mg protein in the right hemisphere.

**Figure 5:**
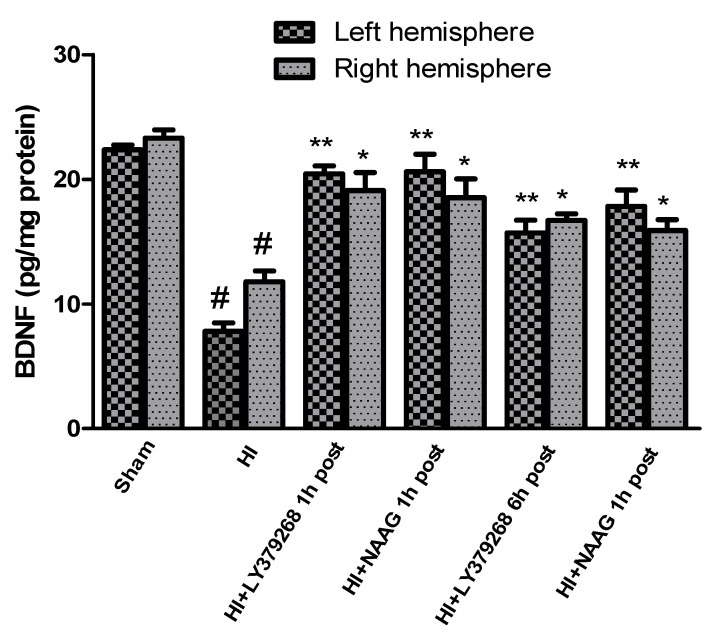
Effect of mGluR2 and mGluR3 agonists injection on BDNF changes after HI. Results are presented as mean ± SEM, n = 6–8; statistically significant differences: * *p* < 0.05, ** *p* < 0.001—different from H-I; # *p* < 0.001, different from sham operated group.

Hypoxia-ischemia significantly decreased BDNF levels in both hemispheres. BDNF level in the left hemisphere decreased after HI to 7.8 pg/mg protein and 11.7 pg/mg protein in the right hemisphere (*p* < 0.001 compared to the sham group for the left and right hemisphere, respectively) (Figure 5).

Early treatment with each of the agonists increased the level of BDNF after the insult almost to the control level. Application of LY379268 1 h after HI increased BDNF level to 20.4 pg/mg and 19.1 pg/mg protein in the left and right hemispheres, respectively (*p* < 0.001 and *p* < 0.05 compared to HI group, for left and right hemispheres, respectively; *p* > 0.05, compared to sham group). LY379268 applied 6 h after HI also increased BDNF expression (15.7 pg/mg protein in the left, 16.7 pg/mg protein in the right hemisphere, *p* < 0.001 for the left and *p* < 0.05 for the right hemisphere); however, this increase was significantly lower than that observed when LY379268 was applied 1 h after HI (*p* < 0.05).

Administration of NAAG 1 h after HI also increased the concentration of BDNF in both hemispheres to the control level; to 20.6 pg/mg protein in the left and 18.5 pg/mg protein in the right, respectively (*p* < 0.001 and *p* < 0.02 compared to HI group, for left and right hemisphere, respectively; *p* > 0.05 compared to sham group). NAAG applied 6 h after HI increased BDNF expression in both hemispheres to 17.8 pg/mg and 15.9 pg/mg protein in the left and right hemispheres, respectively (*p* < 0.001 and *p* < 0.05 for the left and right hemisphere, respectively). However, the level of BDNF was still significantly lower than the control (*p* < 0.01 for both hemispheres).

## 4. Discussion

Our study shows that stimulation of mGluR2/3 in a rat model of neonatal HI shortly after insult not only reduces brain infarct volume but also decreases the apoptosis process and preserves neuroprotective processes.

LY379268, an agonist used in our investigations, is usually used as a highly selective agonist of mGluR2/3; however, it presents two times higher selectivity for mGluR2 than mGluR3. Moreover, it has been shown in knock-out mice, where the lack of mGluR2 or mGluR3 is compensated by an increased level of the remaining receptor, that LY379268 did not show positive action in mGluR2 knock-out mice in two mouse models [42,43]. These findings indicate that actions of LY379268 are mainly mediated by the mGluR2. NAAG is known as an endogenous neurotransmitter localized to neurons with a high affinity for mGluR3, with only weak effects on NMDA receptors [44,45]. Therefore, in the experiments presented in this paper, we used LY379268 as an agonist of mGluR2 and NAAG as a specific mGluR3 agonist.

Presented results confirmed observations from our previous experiments, showing that administration of LY379268 or NAAG not only shortly after HI but also up to 24 h before HI attenuated neurodegeneration [7,34,35].

The equilibrium between Bcl-2 and Bax in HI conditions is significantly disrupted and determines apoptosis induction [46]. In our previous studies, we showed that pretreatment with LY379268 or NAAG significantly reduced HI-induced changes in Bcl-2 and Bax expression inhibiting apoptotic processes [8]. The results presented in this paper show that application of mGluR2/3 agonists in a short time after HI results not only significantly decrease in pro-apoptotic Bax expression but also diminishes HtrA2/Omi expression increased by the insult. This indicates that stimulation of mGluR2/3 up to 6 h after HI may inhibit caspase-dependent and -independent apoptosis.

Our previous studies have shown that activation of mGluR2 or mGluR3 in a therapeutic window determined after HI, which overlaps times used in present studies, significantly reduces oxidative stress evoked by HI [7]. The observed anti-apoptotic effect of LY379268 and NAAG is probably associated with maintaining a good condition of mitochondria, as a result of decreased oxidative stress.

HIF-1 is considered a regulator of both pro-survival and pro-death responses in the central nervous system. There are two phases of HIF-1a activation after cerebral ischemia [47]. The first phase was observed immediately after injury and lasted up to 12 h, when the upregulation of most pro-death genes was detected, and the second phase of HIF-1a activation was characterized by the expression of most pro-survival genes [47,48].

However, neonatal HI, characterized by severe hypoxia and excessive production of ROS, may trigger pro-death responses induced by cumulative HIF-1α activation that dominates over its pro-survival action [22]. Therefore, the increased level of HIF-1α observed in our experiments suggests its pro-apoptotic actions [49]. Administration of LY 379268 or NAAG up to 6 h after HI significantly decreases the level of HIF-1α. A similar effect was observed in our previous experiments, where LY379268 or NAAG pretreatment was accompanied by a decrease in HIF-1α concentration and a decrease in apoptotic damage, suggesting that activation of mGluR2/3 receptors before HI inhibits HIF-1α accumulation during the early phase of apoptosis development [8]. Present results suggest that the same mechanism is activated by the application of LY379268 or NAAG in a short time after HI.

Hypoxia-ischemia conditions activate astrocytes’ synthesis and secretion of many proteins including cytokines and neurotrophic factors, which have an opposite role in the development of ischemic brain damage. Expression of the factors such as interleukin-10 (IL-10) nerve growth factor (NGF), BDNF, and GDNF, can delay HI-induced damage to neurons [24]. GDNF has been reported to have not only anti-apoptotic but also anti-autophagic effects, while BDNF promotes neuronal survival through inhibiting apoptosis, inflammation processes, and promotion of neuronal regeneration [32,50]. It was found that the increased endogenous expression of GDNF may play an important role in the protection of damaged ischemic nerve cells. GDNF with its trophic effects on neurons has been shown to have neuroprotective effects after ischemic brain injury [51].

Kurokawa et.al. [52] showed that the number of TUNEL- and caspase-2-positive cells were lower in the BDNF-treated group in a rat model of retinal ischemia-reperfusion injury. It was also shown that BDNF protects the brain from ischemic injury by modulating local inflammation in rats [53] and reduces apoptosis in neonatal hypoxia-ischemia [14].

The interplay among neurotrophic factors and the mGlu2/3 receptors signaling system has been suggested as a possible mechanism involved in neuroprotection. It was shown that activation of the mGluR2/3 receptors by application of LY379268 increased BDNF expression in mouse brain [29,54,55], whereas activation of mGluR2/3 receptors resulted in an increase in GDNF expression in mouse brain [56]. In contrast to the rare expression of GDNF in adult rat brains, developing neonatal brains show broad expression in a wide subpopulation of neuronal and non-neuronal cells [57]. A similar pattern of GDNF expression has been observed in the developing human fetal brain [58]. The significant developmental change in GDNF protein expression and GDNF concentration in the cerebrospinal fluid of rats aged 1 to 21 days [59] suggests a critical role of GDNF in neuronal differentiation and survival in the developing brain.

Hypoxic-ischemic conditions increase GDNF expression, which reaches its peak 48 h after insult [24,27]; GDNF increased expression measured 7 days after HI was observed mostly in the ipsilateral hemisphere [60].

In our study, HI caused a significant increase in GDNF concentration in both hemispheres; the level of GDNF after administration of LY379268 or NAAG 1 h or 6 h after HI was still high but significantly decreased compared to the HI group. The study by Ikeda et al. [59] demonstrated that increase in GDNF in the neonatal rat brain injury after HI has a bimodal character. The first increase in GDNF is of neuronal origin, and the later one is probably related to progressive astrogliosis that occurs in response to injury. In our experiments, GDNF expression was measured in a short time after HI; therefore, we believe that the neuroprotective effect of activation mGluR2/3 on GDNF expression observed in our study is connected with the protection of neurons by decreasing glutamate release; however, this needs more investigation.

Hypoxia-ischemia significantly decreases the level of BDNF in many regions of the brain, including the cortex and hippocampus [26,61]. It was shown that increasing BDNF expression was beneficial for the neuronal survival after HI through its anti-apoptotic, anti-inflammatory, and anti-neurotoxicity effects, as well as promotion of neural regeneration [32,62,63]. Therefore, factors that maintain the BDNF on an appropriate level may become therapeutic tools.

In our study, HI caused a significant decrease in BDNF concentration in both hemispheres, and administration of LY379268 or NAAG 1 h after HI restored the BDNF level close to control. Both mGluR2/3 agonists applied 6 h after HI also efficiently, but to a lesser extent, inhibited HI and evoked a decrease in BDNF level.

It is believed that the main neuroprotective effect of LY379268 administration is to inhibit the release of glutamate and prevent excitotoxicity. LY379268 neuroprotective effect was shown in the global model of ischemia in gerbils and newborn rats after HI [4,64]. Cai et al. [41] also suggested that the neuroprotective effect of NAAG is largely associated with the activation of the mGlu2/3 receptor.

Our results show that activation of mGluR2/3 receptors shortly after HI protects against HI-induced brain damage. Combine the results presented in this paper with our previous results, where we showed that application of LY379268 or NAAG shortly after HI significantly reduces oxidative stress [7], indicated on the complex neuroprotective effect of mGluR2/3 activation. In our opinion, inhibition of excessive glutamate release and decrease in its extracellular concentration is the main mechanism involved in the observed patterns of neuroprotection.

The fact that application of both mGluR2 and mGluR3 agonists produced comparable results allows the guess that both types of receptors may be a potential target for early treatment in neonatal hypoxia-ischemia.

## Data Availability

The data presented in this study are available in this manuscript.
